# Synthesis and antioxidant activity of some 1-aryl/aralkyl piperazine derivatives with xanthine moiety at N4

**DOI:** 10.1080/13102818.2014.979978

**Published:** 2014-11-17

**Authors:** Lily Andonova, Dimitrina Zheleva-Dimitrova, Maya Georgieva, Alexander Zlatkov

**Affiliations:** ^a^ Department of Pharmaceutical Chemistry, Faculty of Pharmacy, Medical University of Sofia, 2 Dunav Street, 1000Sofia, Bulgaria; ^b^ Department of Pharmacognosy and Pharmaceutical Botany, Faculty of Pharmacy, Medical University of Sofia, 2 Dunav Street, 1000Sofia, Bulgaria

**Keywords:** antioxidant activity, 1-aryl/aralkyl piperazines, xanthine

## Abstract

Piperazine nucleus is one of the most important heterocyclic systems exhibiting remarkable pharmacological activities. Thus, in the current study six new aryl/aralkyl substituted piperazine derivatives, containing methylxanthine moiety were synthesized and their structures were confirmed by IR and ^1^H NMR analysis. All compounds were *in vitro* screened for their activity as antioxidants using DPPH (2,2′-Diphenyl-1-picrylhydrazyl), ABTS (2,2′-azinobis-(3-ethylbenzo thiazine-6-sulfonic acid)) and FRAP (ferric reducing/antioxidant power) methods. The antioxidant activity of the studied compounds against lipid peroxidation was also measured. The highest antioxidant activity was demonstrated by compound 3c. It is obvious that the presence of a hydroxyl group in the structure is essential for the antioxidant properties and should be taken into consideration in further design of structures with potential antioxidant properties.

## Introduction

The cellular injury caused by oxidative stress and excess of free radicals such as reactive oxygen species has been associated with ageing and great number of clinical disorders including age-associated disorders such as neurodegenerative diseases. For example, Parkinson's disease, which is the second most common neurodegenerative disorder after Alzheimer's disease (AD), has been linked to increased levels of oxidative stress.[1,2] Also oxidative stress has been postulated to have an important role in the pathogenesis of schizophrenia, bipolar disorder and major depression.[3,4] It is also discussed, that the radical production and lipid peroxidation are involved in the pathogenesis of other diseases, including atherosclerosis, cardiac and cerebral ischemia, carcinogenesis, diabetes, alcohol induced liver disease, ulcerative colitis and rheumatic disorders.[5–7]

Antioxidant-based drugs and formulations for the prevention and treatment of complex diseases like atherosclerosis, stroke, diabetes, AD, cancer and some psychiatric disorders have appeared during the last decades.[8,9] This has attracted a great deal of research interest in the field of antioxidants.

Piperazine nucleus is one of the most important heterocycles exhibiting remarkable pharmacological activities. Piperazine is consisting of a six-membered ring containing two opposing nitrogen atoms. Slight change in substitution pattern in piperazine nucleus causes distinguishable difference in their pharmacological activities, such as antipsychotic, anticonvulsant, antiarrhythmic, antimicrobial, antimalarial, citotoxic and antioxidant effects.[10–12]

Thus, in this study some new aryl/aralkyl substituted piperazine derivatives, containing methylxanthine moiety were synthesized and their antioxidant activity was established.

## Materials and methods

### Materials

Synthetic grade chemicals procured from Acros organics, Belgium, were used for the synthesis of the target compounds, as received. Non-commercially available intermediates required for the synthesis of novel derivatives of arylpiperazine were prepared according to the literature procedures without modifications: 1-(3-iodopropyl)-3,7-dimethylxanthine.[13] The completion of reactions and the purity of the final products was monitored and confirmed through thin layer chromatography (TLC) using Kieselgel 60 F_254_ plates (Merck) and solvent system: ethanol:chloroform:acetone (4:3:3 ). The spots were detected at UV_254_ nm.

The reagents: 2,2′-Diphenyl-1-picrylhydrazyl (DPPH), 2,2′-azinobis-(3-ethylbenzo thiazine-6-sulfonic acid) (ABTS), sulfanilamide, 6-hydroxy-2,5,7,8-tetramethylchroman-2-carboxylic acid (Trolox), 2,4,6-tripyridyl-s-triazine (TPTZ), ferric chloride × 6H_2_O, sodium acetate and potassium persulphate were purchased from Sigma-Aldrich. All the other chemicals including the solvents were of analytical grade.

### Apparatus

Melting points were determined on an electrothermal apparatus (Büchi 535, Switzerland) in an open capillary tube and are not corrected. The IR spectra 400–4000 cm^−1^ were recorded on an FT-IR Nicolet iS10 spectrophotometer (Thermo Scientific, USA). The ^1^H NMR spectra were measured on a Bruker Avance DRX 250 (250 MHz) spectrometer (Germany). ^1^H NMR spectra were measured for approximately 0.03 mol/L while using CDCl_3_ and D_2_O as solvents and chemical shifts, and were expressed as δ values in ppm against TMS as an internal standard. All names were generated by using structure-to-name and name-to-structure algorithms included with ChemBioDraw Ultra 11.0 (CambridgeSoft). Antioxidant assays were carried out using a Shimadzu UV-1203 spectrophotometer (Japan).

### Synthetic procedures for obtaining of the target compounds 3a–f

#### General procedure

A mixture of 1-(3-iodopropyl)-3,7-dimethyl-1H-purine-2,6(3H,7H)-dione (1, 1 mmol), the appropriate 1-substituted piperazine (2a–f, 1 mmol) and triethylamine (1 mmol) in acetone (10 mL) was refluxed till exhaustion of the starting products. The progress of the reaction was monitored by TLC. Then the acetone was evaporated to dryness. The reaction mixture was cooled overnight if it was necessary. The precipitated crude product was filtered off and purified by recrystallization from *i*-propanol.

#### 1-(3-(4-benzylpiperazin-1-yl)propyl)-3,7-dimethyl-1H-purine-2,6(3H,7H)-dione (3a)

Reaction time 10 hours. Yield: 77%, melting point (m.p.) 191–193 °C, Rf = 0.45, IR: 3070 (CH aromatic), 2911–2833 (CH_3_ and CH_2_), 1702 (CO xanthine), 1654 (CO xanthine), 1602 (C = N xanthine), 1552 (C = C xanthine), 762 (mono-substituted aromatic ring), ^1^H–NMR (CDCl_3_, δ, ppm): 7.54 (s, 1H, C^8^–H); 7.36–7.37 (m, 4H, aromatic ring); 7.27 (bs, 1H, aromatic ring); 4.09 (t, 2H, N^1^(xanthine)–CH_2_); 3.98 (s, 3H, N^7^–CH_3_); 3.84 (bs, 2H, N^1^(piperazine)–CH_2_); 3.56 (s, 3H, N^3^–CH_3_); 3.03 (bs, 8H, piperazine); 2.17 (s, 2H, N^1^(xanthine)–CH_2_–CH_2_), 1.26, 1.22 (d, 2H, CH_2_–N^1^(piperazine), J = 8.43 Hz).

#### 1-(3-(4-(4-fluorophenyl)piperazin-1-yl)propyl)-3,7-dimethyl-1H-purine-2,6 (3H,7H)-dione (3b)

Reaction time 10 hours. Yield: 67%, m.p. 156–158 °C, Rf = 0.64, IR: 3054 (CH aromatic), 2966–2817 (CH_3_ and CH_2_), 1709 (CO xanthine), 1651 (CO xanthine), 1604 (C = N xanthine), 1577 (C = C xanthine), 1143 (C–F), 820 (1,4-disubstituted aromatic ring), ^1^H–NMR (CDCl_3_, δ, ppm): 7.51 (s, 1H, C^8^–H); 6.96 (t, 2H, aromatic ring); 6.85–6.88 (m, 2H, aromatic ring); 4.10 (t, 2H, N^1^(xanthine)–CH_2_); 3.99 (s, 3H, N^7^–CH_3_); 3.58 (s, 3H, N^3^–CH_3_); 3.11 (s, 4H, piperazine); 2.63 (s, 4H, piperazine); 2.54 (bs, 2H, CH_2_–N^1^ piperazine), 1.41 ( t, 2H, N^1^(xanthine)–CH_2_–CH_2_).

#### 1-(3-(4-(4-hydroxyphenyl)piperazin-1-yl)propyl)-3,7-dimethyl-1H-purine-2,6 (3H,7H)-dione (3c)

Reaction time 10 hours. Yield: 93%, m.p. 263–264 °C with decomposition, Rf = 0.43, IR: 3202 (Aryl-OH), 3075 (CH aromatic), 2926–2815 (CH_3_ and CH_2_), 1701 (CO xanthine), 1657 (CO xanthine), 1609 with shoulder 1595 (C˭N xanthine), 1550 (C˭C xanthine), 1359 (δOH), 1186 (C–OH), 828 (1,4-disubstituted aromatic ring), ^1^H–NMR (D_2_O, δ, ppm): 8.39 (s, 1H, OH-aromatic ring) 7.85 (s, 1H, C^8^–H); 6.97–6.85 (m, 2H, aromatic ring); 6.85–6.88 (m, 2H, aromatic ring); 4.04 (t, 2H, N^1^(xanthine)–CH_2_); 3.89 (s, 3H, N^7^–CH_3_); 3.48 (s, 3H, N^3^–CH_3_); 3.34, 3.28 (d, 8H, piperazine, J = 13.08); 3.13 (s, 2H, N^1^(xanthine)–CH_2_–CH_2_), 2.06 (bs, 2H, CH_2_–N^1^(piperazine).

#### 1-(3-(4-(2-methoxyphenyl)piperazin-1-yl)propyl)-3,7-dimethyl-1H-purine 2,6 (3H,7H)-dione (3d)

Reaction time 5 hours. Yield: 75%, m.p. 177–179 °C, Rf = 0.56, IR: 3125 (CH aromatic), 2931–2817 (CH_3_ and CH_2_), 2817 (O–CH_3_), 1694 (CO xanthine), 1652 (CO xanthine), 1602 (C˭ N xanthine), 1548 (C˭C xanthine), 1454 (δCH_3_ O–CH_3_), 1154 (C–O–C), 745 (1,2-disubstituted aromatic ring), ^1^H–NMR (CDCl_3_, δ, ppm): 7.50 (s, 1H, C^8^–H); 7.00–6.97 (m, 1H, aromatic ring); 6.93–6.90 (m, 2H, aromatic ring); 6.86–6.84 (m, 1H, aromatic ring); 4.10 (t, 2H, N^1^(xanthine)–CH_2_); 3.99 (s, 3H, N^7^–CH_3_); 3.85 (s, 3H, OCH_3_–aromatic ring); 3.58 (s, 3H, N^3^–CH_3_); 3.06 (bs, 4H, piperazine); 2.66 (bs, 4H, piperazine); 2.53 (t, 2H, CH_2_–N^1^(piperazine), 1.90 (t, 2H, N^1^(xanthine)–CH_2_–CH_2_).

#### 1-(3-(4-(4-methoxyphenyl)piperazin-1-yl)propyl)-3,7-dimethyl-1H-purine-2,6(3H,7H)-dione (3e)

Reaction time 13 hours. Yield: 64%, m.p. 154–156 °C, Rf = 0.58, IR: 3120 (CH aromatic), 2946–2813 (CH_3_ and CH_2_), 2813 (O–CH_3_), 1698 (CO xanthine), 1651 (CO xanthine), 1604 (C˭N xanthine), 1548 (C˭C xanthine), 1456 (δCH_3_ O–CH_3_), 1125 (C–O–C), 844 (1,4-disubstituted aromatic ring), ^1^H–NMR (CDCl_3_, δ, ppm): 7.50 (s, 1H, C^8^–H); 6.90–6.88 (m, 2H, aromatic ring); 6.84–6.82 (m, 2H, aromatic ring); 4.09 (t, 2H, N^1^(xanthine)–CH_2_); 3.99 (s, 3H, N^7^–CH_3_); 3.76 (s, 3H, OCH_3_-aromatic ring); 3.58 (s, 3H, N^3^–CH_3_); 3.06 (bt, 4H, piperazine); 2.62 (bt, 4H, piperazine); 2.52 (t, 2H, CH_2_–N^1^(piperazine), 1.90 ( t, 2H, N^1^(xanthine)–CH_2_–CH_2_).

#### 1-(3-(4-(bis(4-fluorophenyl)methyl)piperazin-1-yl)propyl)-3,7-dimethyl-1H-purine-2,6 (3H,7H)-dione (3f)

Reaction time 5 hours. Yield: 60%, m.p. 144–146 °C, Rf = 0.74, IR: 3070 (CH aromatic), 2962–2825 (CH_3_ and CH_2_), 1703 (CO xanthine), 1655 (CO xanthine), 1602 (C˭N xanthine), 1551 (C˭C xanthine), 1223 (C–F), 826 (p- di-substituted aromatic ring), ^1^H–NMR (CDCl_3_, δ, ppm): 7.53 (s, 1H, C^8^–H); 7.35–7.27 (m, 4H, aromatic ring); 7.02–6.93 (m, 4H, aromatic ring); 4.36 (s, 1H, N^1^(piperazine)–CH); 4.08 (t, 2H, N^1^(xanthine)–CH_2_); 3.96 (s, 3H, N^7^–CH_3_); 3.56 (s, 3H, N^3^–CH_3_); 3.14 (t, 4H, piperazine); 2.94 (bs, 4H, piperazine); 2.33 (t, 2H, CH_2_–N^1^(piperazine), 1.48 (t, 2H, N^1^(xanthine)–CH_2_–CH_2_).

### Antioxidant assays

#### DPPH radical scavenging activity

Free radical scavenging activity was measured by using the DPPH method.[14] Different concentrations (6.25–1000 μmol/L) (400 μL) of compounds in methanol were added to the 400 μL methanol solution of DPPH (0.5 mmol/L). After 30 min incubation at room temperature, their absorptions were read at 517 nm against a control sample containing the methanol solution of DPPH. Percentage of the DPPH radicals scavenged by the studied concentration was calculated according to equation:
(1) 
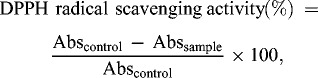
where Abs_control_ is the absorbance of DPPH radical in methanol, Abs_sample_ is the absorbance of DPPH radical solution mixed with sample.

IC_50_ value (concentration of sample where the absorbance of DPPH decreases 50% with respect to the absorbance of blank) of the sample was determined. Butylated hydroxytoluene (BHT) was used as positive control. All determinations were performed in triplicate (*n* = 3).

#### ABTS-radical scavenging assay

For ABTS assay, the procedure followed the method of Arnao et al. [15] with some modifications.[14] The stock solutions included 7 mmol/L ABTS solution and 2.4 mmol/L potassium persulphate solution. The working solution was then prepared by mixing the two stock solutions in equal quantities and allowing them to react for 14 hours at room temperature in dark. The solution was then diluted by mixing 2 mL ABTS solution with 50 mL methanol to obtain an absorbance of 0.305 ± 0.01 units at 734 nm using a spectrophotometer. A fresh ABTS solution was prepared for each assay. Different concentrations (400 μL) of compounds were allowed to react with 400 μL of the ABTS solution and the absorbance was taken at 734 nm after 5 min. The capability to scavenge the ABTS radical was compared with that of BHT and was calculated using the following equation:
(2) 
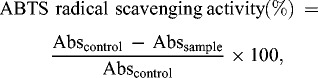
where Abs_control_ is the absorbance of ABTS radical in methanol; Abs_sample_ is the absorbance of an ABTS radical solution mixed with sample.

IC_50_ value (concentration of sample where the absorbance of ABTS decreases 50% with respect to the absorbance of blank) of the sample was determined. BHT was used as positive control. All determinations were performed in triplicate (*n* = 3).

#### Ferric reducing/antioxidant power (FRAP)

The FRAP assay was done according to the method described by Benzie and Strain [16] with some modifications.[14] The stock solutions included 300 mmol/L acetate buffer (3.1 g C_2_H_3_NaO_2_ × 3H_2_O and 16 mL C_2_H_4_O_2_), pH 3.6, 10 mmol/L TPTZ solution in 40 mmol/L HCl and 20 mmol/L FeCl_3_ × 6H_2_O solution. The fresh working solution was prepared by mixing 25 mL acetate buffer, 2.5 mL TPTZ solution and 2.5 mL FeCl_3_ × 6H_2_O solution and then warmed at 37 °C before using. 0.15 mL of compound in methanol was allowed to react with 2.8 mL of the FRAP solution for 30 min in the dark condition. Readings of the coloured product (ferrous tripyridyltriazine complex) were then taken at 593 nm. Results are expressed in μg Trolox equivalent (μM TE/mmol/L]). BHT was used as reference. All determinations were performed in triplicate (*n* = 3).

#### Determination of antioxidant activity in linoleic acid system by the ferric thiocyanate (FTC) method

The antioxidant activity of studied compounds against lipid peroxidation was measured through ammonium thiocyanate assay, as described by Takao et al., [17] with some modifications.[20] The reaction solution, containing 0.2 mL of compound (1 mmol/L in methanol), 0.2 mL of linoleic acid emulsion (25 mg/mL in 99 % ethanol) and 0.4 mL of 50 mmol/L phosphate buffer (pH 7.4), was incubated in the dark at 40 °C. A 0.1 mL aliquot of the reaction solution was then added to 3 mL of 70 % (v/v) ethanol and 0.05 mL of 30% (w/v) ammonium thiocyanate. Precisely 3 min after the addition of 0.05 mL of 20 mmol/L ferrous chloride in 3.5% (v/v) hydrochloric acid to the reaction mixture, the absorbance of the resulting red colour was measured at 500 nm. Aliquots were assayed every 24 hour until the day after the absorbance of the control solution (without compound) reached maximum value. BHT (1 mmol/L) was used as a positive control. All determinations were performed in triplicate (*n* = 3).

## Results and discussion

### Chemistry

**Scheme 1.**
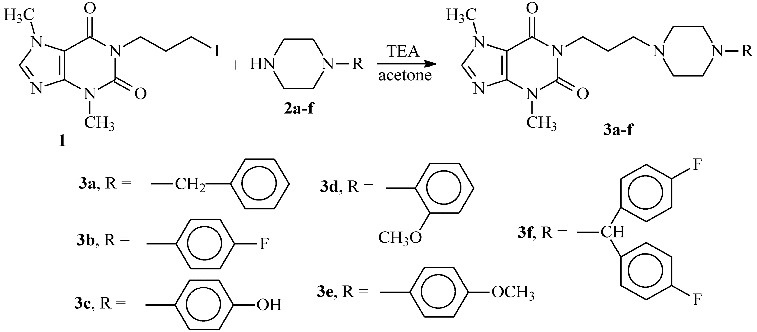
General synthesis of the target compounds 3a–f.

The target compounds have been synthesized according to the above-presented synthetic procedure. The general synthetic scheme is presented in Scheme 1. The readily available compound 1-(3-iodoropropyl)-3,7-dimethylxanthine (1) was aminated with 1-aryl/aralkylpiperazines (2a–f) in the presence of triethylamine to give the target compounds 3a–f in high yields. In this reaction scheme, it is possible to use 1-(3-chloropropyl)-3,7-dimethylxanthine as the starting compound instead of 1. When it was used as a starting material to obtain 3a–f, their yield was found to be remarkably lower and the reaction time about two times longer. This was probably due to low reactivity of this compound in comparison with 1. We also found that if there is no protection from the atmospheric moisture whilst obtaining the target compounds from both of 1 and 1-(3-chloropropyl)-3,7-dimethylxanthine, the hydrolysis took place and the yield of the new compounds decreased. The hydrolysis product is 1-(3-hydroxypropyl)-3,7-dimethylxanthine (13) and was detected by TLC.

All the synthesized compounds were freely soluble in chloroform, dichloromethane, dimethylformamide (DMF) and dimethyl sulfoxide (DMSO), but insoluble in non-polar solvents like *n*-hexane, except 3c which is soluble only in boiling water. The structures of the synthesized compounds were confirmed by IR and ^1^H NMR. The results were consistent with the assigned structures.

The FT-IR spectra of the studied compounds 3a–f in the region of the 4000–400 cm^−1^ exhibit several characteristic bands. Vibrations at 3054–3125 cm^−1^ belong to stretching vibrations of aromatic C–H bonds. The stretching vibration of phenolic group in the spectrum of 3c appears at 3202 cm^−1^. The two strong bands at about 1680–1702 cm^−1^ are ascribable to the stretching vibration of two carbonyl groups in the xanthine ring. The band at about 1550 cm^−1^ belongs to the stretching vibration of C˭C bonds in xanthine ring.

More detailed information about the structure of compounds 3a–f was provided by the ^1^H NMR spectra. The ^1^H NMR spectra of the compounds 3a–f showed the signals of the respective protons of the synthesized compounds, which were verified on the basis of their chemicals shifts, multiplicities and coupling constants. Thus, the strong singlets at 3.48\3.58 and 3.89\3.99 ppm in the spectrum of the studied compounds correspond to *N*-methyl protons at positions 3 and 7. The signal of the proton at position 8 appears at 7.50\7.54 ppm as strong singlet. The signals of methylene protons from the benzyl side chain at position 1 form strong singlet at 4.80 ppm. The signals of the aromatic protons from substituted benzyl side chain in the spectra of 3b–e correspond to complicated multiplets between 6.82 and 6.97 ppm, while the same protons in unsubstituted benzyl moiety (3a) appear as multiplet 7.27 and 7.37 ppm. The aromatic protons in benzhydryl group in 3f form multiplet at 7.27 and 7.35 ppm. The methylene proton from the benzhydryl residue forms a singlet with weak to medium intensity at 4.36 ppm. The integral curves correspond to the exact number of the protons. The values of the chemical shifts of the protons registered by ^1^H NMR spectra were compared with simulated values.[18–21] Due to an impossibility to render an account of influence of the solvent, we observed only small deviations of the computed values from the experimental values. Regardless, the simulated ^1^H NMR spectra are in good correlation with experimental ones.

### Antioxidant assays

In this paper, we apply four common methods [14–17] for the determination of free radicals scavenging activity, total antioxidant capacity and antioxidant activity against lipid peroxidation to both lipophilic and hydrophilic antioxidants in an attempt to establish the most appropriate one for evaluation of possible antioxidant effects, demonstrated from the newly synthesized arylpiperazine derivatives. The described methods were chosen for their user-friendly mechanisms for antioxidant activity determination since they require a simple machine like a spectrophotometer, which is commonly available in most laboratories.

**Figure 1.**
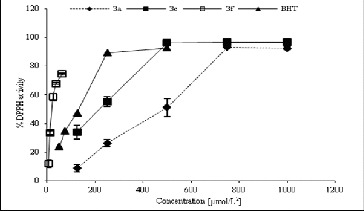
DPPH radical scavenging activity of studied compounds 3a, 3c, 3f and BHT.

**Figure 2.**
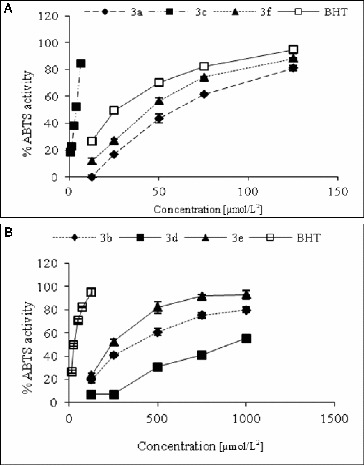
ABTS-radical scavenging activity of studied compounds (a) 3a, 3c, 3f and BHT and (b) 3b, 3e, 3d and BHT.

The free radicals scavenging activity was measured by using DPPH and ABTS methods with slight modifications.[20] The inhibitory effect of different concentrations of compounds (6.25–1000 μmol/L^2^) on DPPH (Figure 1) and ABTS (Figure 2) was determined by recording the absorbance of the reaction mixture at 517 and 734 nm, respectively. The IC_50_ values (concentration of sample where the absorbance of DPPH and ABTS decreases 50% with respect to the absorbance of blank) of the samples were determined.

The applied FRAP assay was done according to the method described previously.[14] The obtained results are expressed in μmol/L Trolox equivalent for mmol/L compound (μmol/L TE/[μmol/L TE/mmol/L]) for 1000 μmol/L^2^ solutions of piperazines.

The corresponding radical scavenging activities against DPPH, ABTS and FRAP of the compounds were compared with those of BHT, used as positive control. All determinations are performed in triplicate (*n* = 3), and the results are expressed as IC_50_ μmol L^−2^ of inhibition in DPPH and ABTS determinations and as mmol/L TE mM^−1^ in FRAP determination. The obtained values are presented in Table 1.

**Table 1.** DPPH, ABTS-radical scavenging and FRAP-activities of studied compounds.

Sample ID	DPPH IC_50_ (μmol/L^2^)	ABTS IC_50_ (μmol/L^2^)	FRAP (μmol/L TE/mmol/L)
3a	371.97	55.87	–
3b	–	345.25	27.22 ± 1.35
3c	189.4224	3.45	173.99 ± 1.50
3d	–	1028.99	–
3e	–	242.48	83.10 ± 1.84
3f	420.57	41.04	–
BHT	113.17	26.29	23.26 ± 0.45

Among the analysed structures, DPPH radical scavenging activity was shown by compounds 3a (IC_50_ 371.97 μmol/L), 3c (IC_50_ 189.42 μmol/L) and 3f (IC_50_ 420.57 μmol/L), with the highest antioxidant activity demonstrated by compound 3c. However, the obtained IC_50_ values of all piperazines were lower than that of BHT (IC_50_ 113.17 μmol/L).

All studied compounds demonstrated ABTS-radical scavenging decreasing in order: 3c (IC_50_ 3.45 μmol/L) > BHT (IC_50_ 26.29 μmol/L) > 3f (IC_50_ 41.04 μmol/L) > 3a (IC_50_ 55.87 μmol/L) > 3e (IC_50_ 242.48 μmol/L) > 3b (IC_50_ 345.25 μmol/L) > 3d (IC_50_ 1028.99 μmol/L).

In the performed FRAP assay, the reduction of ferric tripyridyl triazine (Fe III TPTZ) complex to ferrous form (which has an intense blue colour) at low pH was monitored by measuring the change in the absorption at 593 nm, which is considered to be directly related to the combined or ‘total’ reducing power of the electron donating antioxidants present in the reaction mixture. Among the synthesized compounds only 3b, 3c and 3e (with p-substitution in phenyl ring) manifest some activity in the FRAP method, while compound 3c demonstrated the highest activity (173.99 ± 1.50 μmol/L TE/mmol/L), followed by 3e (83.10 ± 1.84 μmol/L TE/mmol/L) and 3b (27.22 ± 1.35 μmol/L TE/mmol/L). However, piperazines 3b, 3c and 3e exhibited stronger activity compared to BHT (23.26 ± 0.45 μmol/L TE/mmol/L). These effects are probably due to the possibility of the analytes to break up the free radical chain by donating a hydrogen atom.

As seen from the presented results for the three discussed methods used for evaluation of the free radicals scavenging activity and FRAP of the newly synthesized structures, the highest antioxidant activity was demonstrated by compound 3c. We believe that this result is due to the presence of hydroxyl group in the structure of this product.

Furthermore, the difference in the chemical structure of the newly obtained compounds will reflect to their relative ability to quench aqueous peroxyl radicals and to reduce the ABTS+, the DPPH free radical and the ferric ion in *in vitro* systems.

Moreover, ABTS, DPPH and FRAP assays gave comparable results for the antioxidant activity measured for all compounds, whereas the ABTS technique was simple, rapidly performed and was applicable for the determination of the oxidative properties for all the analysed structures. An additional advantage of the ABTS to DPPH is that the reaction is more rapid, whereas the DPPH reaction takes much longer. Therefore, it would be an appropriate technique for antioxidant evaluation of structural derivatives with piperazine residue.

**Figure 3.**
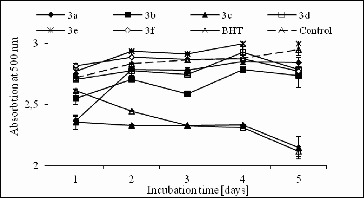
Antioxidant activity of structures 3a–f in the linoleic acid system 1 μmol/L^2^.

In this study, the antioxidant activity of the studied compounds (1000 μmol/L^2^) against lipid peroxidation was also measured through ammonium thiocyanate assay, as described previously.[17] The inhibition of lipid peroxidation of the analysed structures (1 mmol/L) was determined in the linoleic acid system using the FTC method (Figure 3). The obtained results revealed that compound 3c demonstrated the highest significant diminution, whereas it hindered the oxidation of linoleic acid for five days with an effect comparable with the BHT effect, used as a control. However, all other analytes did not show significant inhibition of lipid peroxidation compared to the control.

## Conclusion

A series of six piperazine like derivatives of 3,7-dimethylxanthine (theobromine) was synthesized. The structures of the new compounds were confirmed by IR and ^1^H NMR. The results were consistent with the assigned structures. All compounds were *in vitro* screened for their activity as antioxidants. It was established that the difference in the chemical structure of the compounds reflects to their relative ability to quench aqueous peroxyl radicals and to reduce the ABTS+, DPPH free radical and ferric ion in *in vitro* systems. For the three discussed methods, the highest antioxidant activity was demonstrated by compound 3c. The antioxidant activity of the studied compounds against lipid peroxidation was also measured. No significant inhibition of lipid peroxidation compared to the control has been observed for the set of compounds, except for compound 3c**,** which demonstrated the highest significant diminution of linoleic acid with an effect comparable with the BHT effect, used as a control. From the performed evaluations, we may conclude that the presence of a hydroxyl group in the structure is essential for the antioxidant properties and should be taken into consideration in further design of structures with potential antioxidant properties.
